# The reward and punishment responsivity and motivation questionnaire (RPRM-Q): A stimulus-independent self-report measure of reward and punishment sensitivity that differentiates between responsivity and motivation

**DOI:** 10.3389/fpsyg.2022.929255

**Published:** 2022-08-10

**Authors:** Nienke C. Jonker, Marieke E. Timmerman, Peter J. de Jong

**Affiliations:** ^1^Department of Clinical Psychology and Experimental Psychopathology, University of Groningen, Groningen, Netherlands; ^2^Department of Psychometrics and Statistics, University of Groningen, Groningen, Netherlands

**Keywords:** reward sensitivity, punishment sensitivity, responsivity, motivation to approach, motivation to avoid

## Abstract

Reward and punishment sensitivity seem important traits in understanding behavior in general and psychopathology in particular. Though the definitions used for reward and punishment sensitivity differentiate between responsivity and motivation, the measures thus far used to assess these constructs do not. Further, specificity of the type of reward (e.g., drugs) and punishment (e.g., spiders) in questionnaires might result in measurement bias especially when examining the relationship with psychopathology. Therefore, we developed a stimulus-independent multidimensional questionnaire of reward and punishment sensitivity that differentiates between responsivity and motivation. This study addresses the psychometric qualities of this newly developed reward and punishment responsivity and motivation questionnaire (RPRM-Q). On the basis of exploratory ordinal factor analysis (*N* = 273) that was used to examine the quality of the initial pool of 39 items, the number of items was reduced to 18. Confirmatory ordinal factor analysis on the remaining items in an independent sample (*N* = 328) supported a 18-item four-factor model, and showed acceptable to good internal reliability. The relationship between the subscales of the RPRM-Q and often used questionnaires was examined in the combined sample (*N* = 601), which showed some first support for the ability of the new questionnaire to differentiate between responsivity and motivation to approach/avoid. The findings indicate that the RPRM-Q might be a helpful instrument to further test the relevance of punishment and reward sensitivity in psychopathology.

## Introduction

Sensitivity to reward (i.e., positive consequences) and punishment (i.e., negative consequences) are considered important determinants of behavior. Behavior that is followed by reward is likely to increase in frequency, whereas behavior that is followed by punishment is likely to decrease in frequency (Thorndike, 1932). For example, if drinking alcohol results in a desirable relaxed feeling it is likely one would drink alcohol again. However, if it results in a hangover it is less likely one would drink alcohol again. Importantly, individuals differ in their sensitivity to rewarding and punishing consequences (Gray, 1970). Thus, some individuals are influenced more by the rewarding and punishing consequences of their behavior than others. If, for example, an individual is relatively sensitive to rewarding consequences it is expected that this individual is more likely to drink alcohol again after a positive experience (such as a relaxed feeling), than an individual who is less sensitive to positive experiences. Similarly, an individual who is relatively sensitive to punishing consequences is expected to be less likely to drink alcohol again after a negative experience (such as a hangover), than an individual who is less sensitive to punishment. Thus, lowered or heightened sensitivity to reward and punishment may be important factors in the development and maintenance of psychopathology. Indeed, a relatively high sensitivity to rewarding cues has been linked to a wide range of externalizing behaviors, such as attention deficit hyperactive disorder (Lopez-Vergara and Colder, 2013), and substance use disorder (Jonker et al., 2014). High sensitivity to punishing cues has been linked to several internalizing behaviors such as social phobia, generalized anxiety (Sportel et al., 2011), and depressive symptoms (Brailean et al., 2014).

Traditionally, reward and punishment sensitivity are often defined within the context of the reinforcement sensitivity theory (Gray, 1970). According to this theory, reward sensitivity is proposed to reflect the sensitivity of the behavioral approach system (BAS), and has for example been defined as: “The BAS is sensitive to rewarding stimuli and assumed to guide approach behaviors” (Glashouwer et al., 2014, p. 97). Punishment sensitivity is proposed to reflect the sensitivity of the behavioral inhibition system (BIS), and has for example been defined as: “People high in punishment sensitivity are assumed to have a highly sensitive BIS-system, easily activated when confronted with punishment and exhibiting stronger inhibitory or avoidant responses compared with people scoring lower on punishment sensitivity” (Vandeweghe et al., 2016b, p. 2). In the revised version of the reinforcement sensitivity theory punishment sensitivity is thought to reflect the sensitivity of the flight-fight-freeze system (Gray and McNaughton, 2000). However, this revision has no impact on the type of behavior that is proposed to reflect punishment sensitivity. Thus, also in this revised theory, punishment sensitivity has been taken to index the response toward punishing stimuli (i.e., punishment responsivity) and the tendency to avoid such stimuli. Reward sensitivity is thought to index the response toward rewarding stimuli (i.e., reward responsivity) and the tendency to approach reward.

All in all, the definitions used for reward and punishment sensitivity in the literature quite consistently incorporate both responsivity and approach/avoidance behavior (Loxton and Dawe, 2001; Colder et al., 2011; Verbeken et al., 2012; Dietrich et al., 2014; Glashouwer et al., 2014; May et al., 2016; Vandeweghe et al., 2016a,b; Matton et al., 2017). Although slightly different definitions have been used, for example definitions that do not incorporate responsivity to reward and punishment (Vandeweghe et al., 2016b). In contrast, available measures do not incorporate, or fail to differentiate between responsivity and approach/avoidance motivation (Carver and White, 1994; Torrubia et al., 2001; Corr, 2016). Although often high responsivity and a strong tendency to approach/avoid will go hand in hand, they represent separate components of reward/punishment sensitivity that under some conditions may vary independently and show differential relationships with particular types of symptoms or problem behaviors. For example, the reward deficiency syndrome theory posits that individuals with a low reward responsivity might compensate for this deficit by overeating or using drugs (Blum et al., 1996; Volkow et al., 2002). Thus, a relatively strong tendency to approach reward may compensate for a relatively low responsivity to rewards. Evidence from the addiction literature also supports the relevance of differentiating between reward responsivity and the motivation to approach reward (Stice et al., 2013; Volkow and Morales, 2015). It has for example been suggested that drug use activates the brain’s reward circuit, resulting in the feeling of being “high.” This feeling of reward influences whether the drug will be used again, with individuals who have a sensitive reward system (i.e., relatively highly reward responsive) being more likely to show recurrent drug use. Repeated drug use is, however, thought to result in drug tolerance, by downregulation of the brain’s reward circuit. The rewarding effect of drug use decreases, and there is a need for larger doses of the drugs to obtain the same rewarding feeling (i.e., relatively high motivation to approach reward) (Volkow and Morales, 2015). Thus, reward responsivity and the motivation to approach reward may differentially influence behavior, and perhaps at different moments in the development of particular types of behavior.

In the following we will first discuss three available measures of reward and punishment sensitivity. The Behavioral Inhibition Scale/Behavioral Activation Scale (BIS/BAS) (Carver and White, 1994) and the sensitivity to punishment and sensitivity to reward questionnaire (SPSRQ) (Torrubia et al., 2001) that have been used most widely, and the more recently developed questionnaire: Reinforcement sensitivity theory of personality questionnaire (RST-PQ) (Corr and Cooper, 2016). Although there are also behavioral measures developed to assess reward and punishment sensitivity (e.g., Derryberry and Reed, 2002; Colder et al., 2011), the focus here will be on self-report measures. Using behavioral tasks has benefits (e.g., people do not require insight), yet it also has disadvantages. When it comes to a construct such as reward and punishment responsivity a behavioral task might not be a good option because these constructs are inherently subjective and critically depend on people’s own interpretation/appreciation. When it comes to motivation to approach reward and avoid punishment this might be different. However, since behavioral tasks often take a lot of time to complete and require specific equipment, a good self-report measure seems an important and helpful tool. Table 1 shows the subscales of the BIS/BAS, the SPSRQ and the RST-PQ and items from these subscales to illustrate the large differences between the questionnaires.

**TABLE 1:** Subscales and items of the BIS/BAS, SPSRQ, and RST-PQ.

Reward sensitivity		
Questionnaire	Subscale	Item from questionnaire
BIS/BAS	Reward responsiveness	When I see an opportunity for something I like I get excited right away When I get something I want, I feel excited and energized
	Reward drive	If I see a chance to get something I want, I move on it right away When I want something I usually go all-out to get it
	Fun seeking	I crave excitement and new sensations I often act on the spur of the moment
SPSRQ	Sensitivity to reward	Does the good prospect of obtaining money motivate you strongly to do some things? Are there a large number of objects or sensations that remind you of pleasant events?
RST-PQ	Reward interest	I am a very active person I am always “on the go”
	Goal-drive persistence	I’m motivated to be successful in my personal life I often overcome hurdles to achieve my ambitions
	Reward reactivity	I am especially sensitive to reward I find myself reacting strongly to pleasurable things in life
	Impulsivity	I often do risky things without thinking of the consequences I think the best nights out are unplanned

**Punishment sensitivity**		
**Questionnaire**	**Subscale**	**Item from questionnaire**

BIS/BAS	Punishment sensitivity	Criticism or scolding hurts me quite a bit I worry about making mistakes
SPSRQ	Sensitivity to punishment	Do you often refrain from doing something because you are afraid of it being illegal? Are you often worried by things that you said or did?
RST-PQ	Fight-flight-freeze	I would be frozen to the spot by the sight of a snake or spider I would run fast if I knew someone was following me late at night
	Behavioral inhibition	I sometimes feel “blue” for no good reason My behavior is easily interrupted

BIS/BAS, Behavioral Inhibition Scale/Behavioral Activation Scale; SPSRQ, Sensitivity to Punishment and Sensitivity to Reward Questionnaire; RST-PQ, Reinforcement Sensitivity Theory of Personality Questionnaire.

The most important difference between the three questionnaires is the type of behavior that is indexed in the subscales. The BIS/BAS has three subscales related to reward sensitivity. Two of them, the reward responsiveness and reward drive subscales, seem in line with the most often used definition of reward sensitivity. The third, the fun seeking subscale, is thought to be a measure of impulsivity or sensation seeking rather than of reward sensitivity and it is suggested that it should be discarded when indexing reward sensitivity (Scheres and Sanfey, 2006). However, often an average of the item scores of all three subscales is used as a measure of reward sensitivity (BAS-total). Thus, when the total score is used the fun seeking items are included. The RST-PQ differentiates between four aspects of reward sensitivity, of which goal-drive persistence and reward reactivity seem in line with the most often used definition of reward sensitivity. In contrast, the SPSRQ has only one reward sensitivity subscale which seems to consist of a mixture of questions about responsivity and approach/avoidance behavior. All three questionnaires do not differentiate between the responsivity and avoidance facets of punishment sensitivity.

Another difference between the questionnaires concerns the content of the questions. In the statements of the BIS/BAS, the type of reward or punishment is often not specified (e.g., “good things,” “something I want”). Yet, the SPSRQ was specifically developed to measure sensitivity to specific rewarding and punishing cues, and asks about a range of specific rewarding and punishing cues such as drugs and social approval (Torrubia et al., 2001). The RST-PQ also often asks about specific cues, such as snakes and barking dogs. Although sensitivity to general as well as specific rewarding and punishing cues are potentially important in determining behavior, the choice of specific cues in the SPSRQ and the RST-PQ complicates interpretation and may cause measurement bias. An example of this can be found in the eating disorder literature. Patients with anorexia nervosa (AN) reported relatively low reward sensitivity on the BIS/BAS (Claes et al., 2006; Jappe et al., 2011), yet relatively high sensitivity to reward on the SPSRQ (Jappe et al., 2011; Glashouwer et al., 2014). The relatively high reward sensitivity on the SPSRQ was found to be due to scores on specifically the items about appearance and interpersonal feedback (Glashouwer et al., 2014). Since patients with AN have a negative body image, and body weight and shape have an undue influence on their self-evaluation (DSM-5) (American Psychiatric Association, 2013), these question might actually be tapping into their punishment sensitivity. The specificity of the items of the RST-PQ might cause similar problems. For example, the questions about specific phobias (e.g., heights or spiders), will cause measurement bias when examining the relationship between punishment sensitivity and symptoms of small animal phobia. Thus, specificity of the rewarding and punishing cues in questionnaires might result in incorrect conclusions about the role of reward and punishment sensitivity in individual’s behavior.

To sum up, the questionnaires that are predominantly used as indices of reward and punishment sensitivity have important limitations. The RST-PQ and the SPSRQ include specific rewarding and punishing cues, which in itself might be important to investigate, yet the wide range that is included has been shown to result in measurement bias. Additionally, the SPSRQ does not differentiate between reward responsivity and motivation to approach reward, and both the SPSRQ and the RST-PQ do not differentiate between punishment responsivity and motivation to avoid punishment. The BIS/BAS is a more general (content independent) measure, however it also does not differentiate between punishment responsivity and motivation (or drive) to avoid punishment.

Because of the limitations of the available measures of reward and punishment sensitivity, there is a clear need for a new questionnaire that measures sensitivity to reward and punishment independent of specific stimuli, and that differentiates between individuals’ responsivity to punishment/reward and their motivation to avoid/approach punishment/reward. Therefore, we developed a new questionnaire in an attempt to resolve the limitations of the available measures that we discussed above. This has resulted in the development of a questionnaire with four subscales, the reward and punishment responsivity and motivation questionnaire (RPRM-Q). To keep it an efficient measure, a short questionnaire with a total of approximately twenty items (five per subscale) was intended. Items are answered on a 5-point scale ranging from “*This does not apply to me at all*” to “*This applies to me completely.*” The current article describes the studies that were carried out to develop and evaluate the final version of the questionnaire. As a first step, the psychometric properties of the questionnaire were examined in a sample of undergraduate students. The exploratory and confirmatory factor analyses of the questionnaire will be reported. In addition, the interrelationships between the four subscales of the new questionnaire were examined. As a second step, the robustness of the psychometric outcome of the first step in a new but relatively similar sample. As a third step, the relation with the two most prominent questionnaires (BIS/BAS and SPSRQ) have been inspected to get an impression of how the RPRM-Q relates to often used reward and punishment sensitivity questionnaires.

## Step 1: Exploratory factor analysis

Based on two pilots a questionnaire of in total 39 items was developed (see Supplementary material 1 for all 39 items). The content of the items was mainly based on literature from the field of reward and punishment sensitivity, and partly on the interpretation of the concepts—as also articulated in the introduction—by the researchers. Two pilots (*N* = 60 and *N* = 126) were set out amongst undergraduate students to give a first impression of whether the items that were intended for the one subscale correlated with each other, and whether the full range of answer options was used. The 39 item questionnaire consisted of 12 items for reward responsiveness, 9 items for motivation to approach reward, 10 items for punishment responsivity, and 8 items for motivation to avoid punishment. More items than needed were included allowing the selection of the best fitting items based on the data of a more appropriately sized sample. The first step was to examine the quality of the items that were designed for each subscale, and to see to what extent we could decrease the number of questions while keeping a good fit.

### Materials and methods

#### Participants

Participants were undergraduate students in an English bachelor program from the University of Groningen. A sample of 273 students (75.1% female) between 17 and 31 years of age (*M* = 20.39, *SD* = 2.03) participated. Initially 297 students participated, however, 24 of them did not correctly answer the control questions (see Procedure), and were therefore excluded. No other in- or exclusion criteria were applied to the sample. Based on the rule of thumb that at least 150 participants are required when factor indicators are normally distributed and there is no missing data we aimed for including at least 200 participants (Muthén and Muthén, 2002).

#### Procedure

The current study was approved by the ethical committee of the psychology department of the University of Groningen (15021). Participants could sign up for an online questionnaire study via the platform of the University, and received study credits for their participation. Participants provided consent for the study online. The RPRM-Q with 39 items was the first questionnaire, followed by the BIS/BAS and the SPSRQ which will be reported on in step 3. At the end of the questionnaire two control questions were included: “This is a control question; click on the most left answering option,” and “This is a control question: click on the most right answering option.” These questions were used to filter out participants who did not answer the questionnaire seriously. The current study additionally served as screening for other studies and therefore contained the Positive And Negative Affect Schedule (Peeters et al., 1996), Center for Epidemiologic Studies Depression Scale (Radloff, 1977), Beck Anxiety Inventory (Beck et al., 1988), Fear Questionnaire (Marks and Mathews, 1979), Restraint scale (RS; Herman and Polivy, 1980), Eating Disorder Examination Questionnaire (EDE-Q; Fairburn and Beglin, 2008), Emotional Eating Scale (Arnow et al., 1995), General Food Craving Questionnaire Trait (GFCQT; Nijs et al., 2007), Alcohol Use (Schippers et al., 2011), and Drinking Motivation Questionnaire (Cooper, 1994).

#### Analyses

No data cleaning or outlier handling was performed on the data. Data is available on https://osf.io/j5x6h/. To account for the ordinal polytomous character of the items, ordinal exploratory factor analyses (EFA) with Oblimin rotation were performed using *Mplus* version 5.2 (Muthen and Muthen, 2007). A step wise analysis plan was followed, to first select per subscale the items of good quality, and subsequently assess for the resulting item set the support for the subscales. First, for each of the four subscales the item set was established. That is, for each subscale, one-factor up to four-factor models were fitted on the items intended for that subscale, to find a set of items that is fitted well by a one-factor model, and that covers the breadth of the pertinent construct. Choices about what items to exclude from a scale were made based on a combination of content and statistical outcome. Thus, at all times a balance was pursued between keeping only qualitative items that produce a good fit, while maintaining a range of questions to keep a broad enough coverage of the pertinent construct. After the decision to exclude an item (or sometimes several at the same step) a new EFA was performed on the remaining items. This process was continued until we reached an acceptable amount of items (preferably four-five), and a good fit (RMSEA < 0.08, and CFI > 0.95) (Hu and Bentler, 1999). Some of the choices are written out below to provide examples but all steps can be viewed in the Supplementary material 2. For sake of completeness we will also report TLI of the final steps.

The final subscale with factor loadings will be reported. Secondly, an EFA with four factors with Oblimin rotation was performed on all selected items, to examine to what extent the four subscales could be distinguished. Internal consistency was assessed with Omega’s coefficient on the basis of the associated one-factor model. Omega coefficient scores under 0.6 are considered poor, between 0.6 and 0.7 questionable, between 0.7 and 0.8 acceptable, between 0.8 and 0.9 good, and above 0.9 excellent. Omega’s coefficient is considered a better indication of internal consistency than the more often used Cronbach’s alpha, since it provides the internal consistency of subscales given that the model is correct, whereas the Cronbach’s alpha provides the lower boundary of the internal consistency (Sijtsma, 2009).

### Results

#### Reward responsivity

Participants completed 12 items that were intended to measure reward responsivity. These items were step-wise reduced to a subscale with four items (see the one-factor EFA column in Table 2). For example, the items “When something good happens, it affects me more strongly than others” and “I become more easily excited by positive outcomes than other people” were deleted, because they formed a factor on their own and they ask specifically about the comparison of people’s own sensitivity with that of other people. Reward responsivity is an automatic process that might not be seen directly in behavior, making a comparison question rather difficult. The step-wise reduction process resulted in a subscale with four items since during all previous steps the fit of the one-factor solution was not good enough (e.g., RMSEA = 0.11 and CFI = 0.98 for the one-factor solution on the last five items). The fit of the final factor solution on the four items was good (RMSEA = 0.00, CFI = 1.00, TFI = 1.00), and the internal consistency of the scale acceptable (Omega’s coefficient = 0.75).

**TABLE 2:** Results of the one-factor EFA per subscale and the final four-factor EFA after Oblimin rotation.

	One-factor EFA	Four-factor EFA			
Question		RR	MR	PR	MP
Winning makes me enthusiastic	**0.53**	**0.47**	0.09	0.07	0.01
Positive outcomes motivate me strongly	**0.88**	**0.87**	0.05	0.03	–0.09
Obtaining rewards affects me strongly	**0.68**	**0.60**	0.08	0.06	0.24
When good things happen to me it affects me strongly	**0.52**	**0.59**	–0.10	–0.04	–0.03
When I want something I usually go all-out to get it	**0.75**	0.11	**0.68**	–0.07	0.01
I go out of my way to get things I want	**0.65**	–0.07	**0.77**	0.08	–0.14
I am more inclined to work hard to get positive outcomes than others	**0.64**	0.02	**0.59**	–0.01	0.13
If I see a chance to get something I want I move on it right away	**0.60**	0.29	**0.42**	–0.18	0.17
I work hard for things that are potentially rewarding for me	**0.61**	0.30	**0.43**	–0.03	0.16
Criticism or scolding hurts me a lot	**0.72**	0.01	0.04	**0.67**	0.10
I feel lousy after doing something wrong	**0.66**	0.18	–0.09	**0.63**	–0.02
When someone points out I did something wrong I feel miserable	**0.84**	–0.09	0.06	**0.89**	–0.03
Receiving punishment affects me strongly	**0.79**	0.11	–0.08	**0.75**	0.03
I feel really bad when something negative happens to me	**0.63**	0.17	0.00	**0.43**	0.30
I do everything in my power to avoid receiving punishment	**0.61**	0.13	0.04	0.12	**0.55**
I go out of my way to avoid unpleasant things happening to me	**0.58**	–0.07	0.16	0.10	**0.51**
I do everything I can to avoid receiving criticism	**0.59**	–0.13	0.01	**0.47**	**0.31**
I avoid things that might have a negative outcome	**0.75**	–0.06	–0.10	0.06	**0.73**

RR, Reward Responsivity; MR, Motivation to approach Reward; PR, Punishment Responsivity; MP, Motivation to avoid Punishment. Factor loadings > 0.3 are printed in boldface.

#### Motivation to approach reward

Participants answered nine questions that were intended to measure motivation to approach reward. After EFA analyses and critical content evaluation a final subscale with five items remained (see the one-factor EFA column in Table 2). The item “I always try to get things I want, even if it means I have to work hard for it” was for example deleted because in retrospect it seemed more a question about perseverance or persistency than it is about motivation to approach reward. The final factor solution of the five items had a good fit (RMSEA = 0.04, CFI = 1.00, TLI = 0.99), and an acceptable internal consistency (Omega’s coefficient = 0.79).

#### Punishment responsivity

Participants answered 10 questions that were intended to measure punishment responsivity. This was reduced to a final subscale with five items (see the one-factor EFA column in Table 2). One of the items that did not end up in the final scale was for example: “Negative outcomes affect me more strongly than others.” In a factor solution with a good fit, this item would end up in a factor on its own. Therefore, and in line with earlier considerations about comparison questions with regard to reward responsivity, this item was deleted. The one-factor model of the final five items showed a good fit (RMSEA = 0.00, CFI = 1.00, TLI = 0.99), and a good internal consistency (Omega’s coefficient = 0.85).

#### Motivation to avoid punishment

The initial questionnaire contained eight questions intended to measure motivation to avoid punishment. The exploratory factor analyses showed that for example the item “I work hard to ensure I will not be rejected” was a factor on its own in good factor solutions. Since in retrospect rejection is a quite specific cue, this item was deleted. The final result was a subscale with four items (see the one-factor EFA column in Table 2). The factor analyses showed a good fit (RMSEA = 0.00, CFI = 1.00, TLI = 0.99), and the subscale had acceptable internal consistency (Omega’s coefficient = 0.73).

#### The reward and punishment responsivity and motivation questionnaire

The final step was an ordinal exploratory factor analysis on the 18 items that were selected in the previous steps. The four-factor solution for these 18 items had a good fit (RMSEA = 0.03, CFI = 0.99, TLI = 0.99). Table 2 shows the factor loadings for all questions. Aside from the good fit, it can be seen that apart from the item “I do everything I can to avoid receiving criticism,” all items had a high loading only on the factor that they were intended to load on.

The item “I do everything I can to avoid receiving criticism” seems to fit better with the punishment responsivity subscale than the motivation to avoid punishment subscale, according to the factor analysis. Further analyses on the item showed that it correlates with two items from the punishment responsivity subscale (*r* = 0.48 with “Criticism or scolding hurts me a lot”; and *r* = 0.50 with “When someone points out I did something wrong I feel miserable”). Although the fit of the factor analyses would improve when this item would be added to the PR instead of the MP subscale, based on its content—it is not about the responsiveness to criticism but about the impact it has on behavior—it was decided to keep it as an item of the Motivation to avoid Punishment subscale.

## Step 2: Confirmatory factor analysis

A second study was performed to evaluate the robustness of the psychometric outcome of the first study in a new but relatively similar sample. Now only the 18 selected items of the questionnaire were administered (see Supplementary material 3 for the questionnaire).

### Materials and methods

#### Participants

Participants were 328 undergraduate students (69.2% female) from an English bachelor program of the University of Groningen. They were between 17 and 34 years of age (*M* = 20.30, *SD* = 2.18). Initially, 341 students participated, however, 13 of them did not correctly answer the control questions and were therefore excluded. No other in- or exclusion criteria were applied to the sample. As for part 1 we aimed for at least 200 participants in the current study (Muthén and Muthén, 2002).

#### Procedure

The current study was approved by the ethical committee of the psychology department of the University of Groningen (16011). Participants could sign up for an online questionnaire study via the platform of the University, and received study credits for their participation. Participants provided consent for the study online. The RPRM-Q with 18 items was the first questionnaire in a larger study. At the end of the questionnaire the same two control questions as in step 1 were included. The RPRM-Q was followed by the BIS/BAS and the SPSRQ which will be reported on in step 3. The questionnaire further contained the Attentional Network Task (Fan et al., 2002), RS (Herman and Polivy, 1980), Perceived Self-Regulatory Success Scale (Fishbach et al., 2003), EDE-Q (Fairburn and Beglin, 2008), GFCQT (Nijs et al., 2007), Clinical Perfectionism Questionnaire (Fairburn et al., 2003), Salience of possible thin and fat self (Dalley, 2016), Approach and Avoidance Temperaments (Elliot and Thrash, 2010), of which most were included for another study and reported on in Jonker et al. (2021).

#### Analyses

No data cleaning or outlier handling was performed on the data. Data is available on https://osf.io/j5x6h/. Ordinal confirmatory factor analysis was performed using *Mplus* version 5.2 (Muthen and Muthen, 2007). A one-factor model was fit for each subscale to assess the quality of items per subscale, and a four-factor model with the items related to a specific subscale, all related to a single factor. Furthermore, internal consistency was assessed with Omega’s coefficient.

### Results

The fit of the one-factor models, and the internal consistency of the subscales are given in Table 3, and factor loadings in Table 4. Results show that the fit of the reward responsivity, motivation to approach reward, and motivation to avoid punishment were good based on the CFI (CFI > 0.95) and of the motivation to approach reward subscale somewhat lower than desired (CFI = 0.91). Based on the RMSEA only the Motivation to avoid Punishment subscale showed a good fit (RMSEA < 0.08). However, it has been suggested that the RMSEA index tends to be too strict at small sample sizes, and is therefore less preferred (Hu and Bentler, 1999). All subscales showed acceptable to good internal consistency.

**TABLE 3:** Fit measures CFI and RMSEA and internal consistency of the four separate subscales.

Subscale	CFI	RMSEA	TLI	Omega’s coefficient
RR	0.99	0.09	0.97	0.76
MR	0.91	0.25	0.82	0.82
PR	0.99	0.11	0.98	0.87
MP	0.99	0.03	1.00	0.79

RR, Reward Responsivity; MR, Motivation to approach Reward; PR, Punishment Responsivity; MP, Motivation to avoid Punishment.

**TABLE 4:** The factor loadings of a one factor model per subscale.

Question	RR			
Winning makes me enthusiastic	0.46			
Positive outcomes motivate me strongly	0.79			
Obtaining rewards affects me strongly	0.77			
When good things happen to me it affects me strongly	0.60			
		**MR**		
When I want something I usually go all-out to get it		0.75		
I go out of my way to get things I want		0.68		
I am more inclined to work hard to get positive outcomes than others		0.60		
If I see a chance to get something I want I move on it right away		0.68		
I work hard for things that are potentially rewarding for me		0.73		
			**PR**	
Criticism or scolding hurts me a lot			0.72	
I feel lousy after doing something wrong			0.70	
When someone points out I did something wrong I feel miserable			0.85	
Receiving punishment affects me strongly			0.70	
I feel really bad when something negative happens to me			0.80	
				**MP**
I do everything in my power to avoid receiving punishment				0.58
I go out of my way to avoid unpleasant things happening to me				0.76
I do everything I can to avoid receiving criticism				0.76
I avoid things that might have a negative outcome				0.67

RR, Reward Responsivity; MR, Motivation to approach Reward; PR, Punishment Responsivity; MP, Motivation to avoid Punishment.

A confirmatory factor analyses with four factors on the 18 RPRM-Q items resulted in a moderate fit (CFI = 0.89, RMSEA = 0.10, TLI = 0.92). Modification indices showed that the fit would be improved by allowing some items to load on more than one factor. For example the item “I go out of my way to get things I want” had a high Modification Index (M.I.) for the Reward Responsivity factor (M.I. = 19.83), and the Motivation to avoid Punishment factor (M.I. = 14.51). We refrained from optimizing based on M.I.s, because such changes yield subscales that are difficult to interpret.

## Step 3: Comparison to existing questionnaires

The final step was to consider the mutual relationships between the four RPRM-Q subscales, and to relate the RPRM-Q subscales to the most often used scales to measure reward and punishment sensitivity, the BIS/BAS and the SPSRQ. The correlations between the subscales of the RPRM-Q are important to examine since we did not want a questionnaire with independent measures, however, they should not measure the same construct either. In other words, subscales should correlate, but only moderately. The comparison with the BIS/BAS and SPSRQ was selected to give an impression of how the new subscales relate to these often used measures. On top of that it allowed to examine whether the BIS/BAS and SPSRQ indeed provide mixed measures of the responsivity and motivation concepts.

Our expectations were that: (1) the reward responsivity (RR) subscale and Motivation to approach Reward (MR) subscale of the RPRM-Q would be related to the reward subscales of both the BIS/BAS and the SPSRQ, (2) the RR subscale of the RPRM-Q would be more strongly related to the Reward Responsivity subscale of the BIS/BAS than to the Reward Drive subscale of the BIS/BAS, (3) the MR subscale of the RPRM-Q would be more strongly related to the Reward Drive subscale of the BIS/BAS than to the Reward Responsivity subscale of the BIS/BAS, (4) the RR and MR subscales of the RPRM-Q would not, or hardly, be related to the punishment sensitivity subscales of the BIS/BAS and SPSRQ, (5) the punishment responsivity (PR) subscale and the motivation to avoid punishment (MP) subscales of the RPRM-Q would be related to the punishment subscales of the BIS/BAS and the SPSRQ, and lastly (6) the PR and MP subscales of the RPRM-Q would not, or hardly, be related to the reward subscales of these questionnaires. Overall moderate correlations were expected.

### Materials and methods

#### Participants

For this analyses the samples of step 1 (*n* = 273) and step 2 (*n* = 328) were combined resulting in a total sample of 601 participants (71.9% females). Participants were between 17 and 34 years old (*M* = 20.34, *SD* = 2.11).

#### Materials

##### The behavioral inhibition scales/behavioral activation scales (BIS/BAS)

This BIS/BAS measures reward (BAS) and punishment (BIS) sensitivity (Carver and White, 1994). The BAS has three subscales; drive, fun seeking, and reward responsiveness. The questionnaire has 20 items which are scored on a four-point Likert scale from “*very true for me*” to “*very false for me*.” In the current study the BIS, which consists of eight items, the BAS- reward responsiveness (BAS-RR), which consists of five items, and the BAS- drive (BAS-Dr), will be reported. Subscale scores are the average scores of the relevant items. The subscales had acceptable internal consistency (Cronbach’s alpha of 0.79, 0.78, and 0.65, respectively).

##### The sensitivity to punishment and sensitivity to reward questionnaire

The SPSRQ contains 24 questions about sensitivity to reward (SR) and 24 questions about sensitivity to punishment (SP) (Torrubia et al., 2001). Participants can answer with either *yes* or *no*. Scores for both subscales are calculated by summing the questions on which participants answered *yes*. Scores can range from 0 to 24 for each subscale and higher scores reflect a higher sensitivity to either reward or punishment. Internal consistency of the RS and PS subscales in the current study were average to good (Cronbach’s alpha of 0.71, and 0.86, respectively).

#### Procedure

In both studies discussed in step 1 and 2, the RPRM-Q was followed by the BIS/BAS and the SPSRQ. The order of the BIS/BAS and the SPSRQ was randomly assigned to prevent order effects.

#### Analyses

Correlational analyses were performed to examine the relation between the subscales of the RPRM-Q, and between the RPRM-Q, and the BIS/BAS or the SPSRQ.

### Results

#### Mutual relationships between the reward and punishment responsivity and motivation questionnaire subscales

As can be seen in Table 5 the correlations between the subscales of the RPRM-Q were mostly in line with what would be expected. The reward subscales showed a moderate correlation, and the punishment subscales showed a moderate correlation. Furthermore, the rest of the correlations were weak.

**TABLE 5:** Correlations between the subscales of the RPRM-Q.

	RR	MR	PR
MR	0.45*	–	–
PR	0.18*	0.03	–
MP	0.19*	0.16*	0.60*

*p < 0.001. RR, Reward Responsivity; MR, Motivation to approach Reward; PR, Punishment Responsivity; MP, Motivation to avoid Punishment.

#### Relation between reward and punishment responsivity and motivation questionnaire and the behavioral inhibition system/behavioral approach system

As can be seen in Table 6, the Reward Responsivity and Motivation to approach Reward subscales of the RPRM-Q showed a moderate correlation to the reward scales of the BIS/BAS. Additionally, the Reward Responsivity scale showed the strongest correlation to the Reward Responsivity subscale of the BIS/BAS, and the Motivation to approach Reward subscale correlated strongest to the Reward Drive subscale of the BIS/BAS. The Reward Responsivity subscale also showed a weak but significant correlation with the BIS subscale.

**TABLE 6:** Correlations between the subscales of the RPRM-Q, the BIS/BAS, and the SPSRQ.

	BIS	BAS_Dr	BAS_RR	RS	PS
**RR**	0.15**	0.30**	0.57**	0.34**	–0.02
**MR**	–0.02	0.64**	0.39**	0.28**	–0.13*
**PR**	0.74**	–0.04	0.20**	0.08	0.58**
**MP**	0.52**	0.12*	0.16**	0.13*	0.49**

** *p* < 0.001, * *p* < 0.01. RR, Reward Responsivity; MR, Motivation to approach Reward; PR, Punishment Responsivity; MP, Motivation to avoid Punishment; BIS, punishment sensitivity of the BIS/BAS; BAS-Dr, Reward drive of the BIS/BAS; BAS-RR, Reward responsivity of the BIS/BAS; RS, Reward Sensitivity of the SPSRQ; PS, Punishment Sensitivity of the SPSRQ.

Both the Punishment Responsivity and the Motivation to avoid Punishment subscales of the RPRM-Q were moderately to strongly related to the BIS scale of the BIS/BAS. This relation was somewhat stronger for the Punishment Responsivity subscale than for the Motivation to avoid Punishment subscale. The Punishment Responsivity subscale and the Motivation to avoid Punishment subscales also showed a weak but significant correlation to the Reward Responsivity subscale of the BIS/BAS, and the Motivation to avoid Punishment subscale to the Drive subscale of the BAS as well.

#### Relation between reward and punishment responsivity and motivation questionnaire and the sensitivity to punishment and sensitivity to reward questionnaire

As can be seen in Table 6, the Reward Responsivity and Motivation to approach Reward subscales of the RPRM-Q showed moderate correlations with the Punishment Sensitivity subscale of the SPSRQ. The Motivation to approach Reward subscale also showed a weak negative correlation to the Punishment Sensitivity subscale of the SPSRQ.

The Punishment Responsivity and Motivation to avoid Punishment subscales showed a moderately strong correlation to the Punishment Sensitivity subscale of the SPSRQ. The Motivation to avoid Punishment also showed a weak but significant correlation to the Reward Sensitivity subscale of the SPSRQ.

## Discussion

The present article describes three steps that were taken to develop a new brief questionnaire to measure the concepts of reward and punishment sensitivity. This questionnaire was developed to resolve the lack of matching between the definitions of reward and punishment sensitivity and their operationalization used in existing questionnaires, and the risk of measurement bias caused by the specificity of the type of reward and punishment in existing questionnaires. The new questionnaire has 18 items and differentiates between responsivity and motivation, resulting in four subscales; reward and punishment responsivity, and motivation to approach reward and avoid punishment. The factor analyses on the subscales showed that the fit of the items was good, with the exception of the motivation to approach reward subscale which showed an acceptable fit. A four-factor CFA in a validation sample of undergraduate students yielded a moderate fit, thereby providing support of the four subscales. Furthermore, the subscales showed acceptable to good internal consistency.

The factor analyses and the correlational analyses showed some first support for the ability of the new questionnaire to differentiate between responsivity and motivation to approach/avoid. This can be seen in the correlations between the subscales, for example reward responsivity and motivation to approach reward were only moderately correlated. It can also be seen in the relation with the questionnaire that does differentiate between these aspects with its reward subscales—the BIS/BAS. The reward responsivity subscale of the RPRM-Q related most strongly to the reward responsivity subscale, and the motivation to approach reward correlated most strongly with the reward drive subscale. The correlational analyses also shows that the thus far most often used questionnaires most likely measures a combination of responsivity and motivation to approach/avoid. The reward sensitivity subscale of the SPSRQ related to both the reward responsivity and the motivation to approach reward questionnaire. Similarly, the punishment sensitivity subscale of both the BIS/BAS and the SPSRQ related to both the punishment responsivity and the motivation to avoid punishment subscales of the RPRM-Q. The RPRM-Q enables future research to empirically examine whether reward and punishment responsivity and motivation are (partly) independently related to behavior and symptoms, and whether these characteristics are differentially involved in various types of, or stages of, psychopathology. This differentiation may help guide clinicians what behavior they should target in therapy.

The current studies used relatively large samples and combined content and statistical outcome in the design of the new questionnaires. There are, however, also some limitations to the current studies. Firstly, the examination of the psychometric properties of the questionnaire is limited to undergraduate students. That is, it has been examined in a sample with a restricted age range and educational level. Future studies should examine the reliability and the fit of the suggested factor structure in the community, as well as in populations with mental or behavioral problems. Secondly, it was only examined how the subscales of the RPRM-Q are related and how the RPRM-Q relates to the BIS/BAS and SPSRS. Future studies should examine convergent and discriminant validity of the RPRM-Q.

## Conclusion

The reward and punishment responsivity and motivation questionnaire (RPRM-Q) was developed to measure both responsivity to and motivation for, reward and punishment, which are critical aspects of the definitions of reward and punishment sensitivity. The current study showed some first support for this differentiation. Future studies should examine the psychometric properties of the questionnaire in broader community samples as well as in clinical populations. Additionally, an important next step is to empirically examine the relationship between reward and punishment responsivity and motivation with specific behavior and symptoms, and whether these characteristics are differentially involved in various types and/or stages of psychopathology.

## Data availability statement

The data analyzed for this study are publicly available. This data can be found here: https://osf.io/j5x6h/.

## Ethics statement

The studies involving human participants were reviewed and approved by the Ethical Committee of the Psychology Department of the University of Groningen. The patients/participants provided their written informed consent to participate in this study.

## Author contributions

NJ: conceptualization, data curation, formal analysis, funding acquisition, investigation, methodology, project administration and writing—original draft, review and editing. MT: conceptualization, methodology, and writing—review and editing. PJ: conceptualization, funding acquisition, methodology, and writing—review and editing. All authors contributed to the article and approved the submitted version.
