# Supplementary material for: The reward and punishment responsivity and motivation questionnaire (RPRM-Q): A stimulus-independent self-report measure of reward and punishment sensitivity that differentiates between responsivity and motivation
Source: Front Psychol. 2022 Aug 10;13:929255. doi: 10.3389/fpsyg.2022.929255 (PMC9404870; doi:10.3389/fpsyg.2022.929255)
Supplement: Supplementary file 1 [file Table_1.DOCX]

This questionnaire consists of 39 items. Please read each statement carefully and think of how much each statement applies to you.

|  |  | **This applies to me completely** | **This applies to me a bit** | **Neutral** | **This does not really apply to me** | **This does not apply to me at all** |
| --- | --- | --- | --- | --- | --- | --- |
| 1. | Winning makes me enthusiastic |  |  |  |  |  |
| 2. | Losing gives me a bad feeling |  |  |  |  |  |
| 3. | When I want something I usually go all-out to get it |  |  |  |  |  |
| 4. | I work harder than others to avoid negative outcomes |  |  |  |  |  |
| 5. | I go out of my way to get things I want |  |  |  |  |  |
| 6. | When I am doing something I like I do not consider the consequences |  |  |  |  |  |
| 7. | Obtaining reward is very important to me |  |  |  |  |  |
| 8. | When I do something poorly it affects me strongly |  |  |  |  |  |
| 9. | I do everything in my power to avoid receiving punishment |  |  |  |  |  |
| 10. | I am more inclined to work hard to get positive outcomes than others |  |  |  |  |  |
| 11. | I really dislike being rejected |  |  |  |  |  |
| 12. | Positive outcomes motivate me strongly |  |  |  |  |  |
| 13. | Obtaining rewards affects me strongly |  |  |  |  |  |
| 14. | When I’m good at something, I like to keep at it |  |  |  |  |  |
| 15. | If I see a chance to get something I want I move on it right away |  |  |  |  |  |
| 16. | When I see something I want I will do everything necessary to obtain it |  |  |  |  |  |
| 17. | If something fun seems to be taken away, I do everything I can to prevent this |  |  |  |  |  |
| 18. | I work hard for things that are potentially rewarding for me |  |  |  |  |  |
| 19. | Criticism or scolding hurts me a lot |  |  |  |  |  |
| 20. | When something unpleasant happens I get pretty “worked up” about it |  |  |  |  |  |

|  |  | **This applies to me completely** | **This applies to me a bit** | **Neutral** | **This does not really apply to me** | **This does not apply to me at all** |
| --- | --- | --- | --- | --- | --- | --- |
| 21. | When something good happens, it affects me more strongly than others |  |  |  |  |  |
| 22. | When I achieve something I want, I feel excited and energized |  |  |  |  |  |
| 23. | I feel lousy after doing something wrong |  |  |  |  |  |
| 24. | I always try to get things I want, even if it means I have to work hard for it |  |  |  |  |  |
| 25. | If it seems that I will lose, I will go to extremes to avoid this |  |  |  |  |  |
| 26. | I go out of my way to avoid unpleasant things happening to me |  |  |  |  |  |
| 27. | I do everything I can to avoid receiving criticism |  |  |  |  |  |
| 28. | When someone points out I did something wrong I feel miserable |  |  |  |  |  |
| 29. | Receiving punishment affects me strongly |  |  |  |  |  |
| 30. | If I can obtain reward I am very motivated to give it my all |  |  |  |  |  |
| 31. | I avoid things that might have a negative outcome |  |  |  |  |  |
| 32. | I feel really bad when something negative happens to me |  |  |  |  |  |
| 33. | Doing things I like makes me very happy |  |  |  |  |  |
| 34. | I can really enjoy nice things |  |  |  |  |  |
| 35. | When good things happen to me it affects me strongly |  |  |  |  |  |
| 36. | Negative outcomes affect me more strongly than others |  |  |  |  |  |
| 37. | I work hard to ensure I will not be rejected |  |  |  |  |  |
| 38. | I like to be rewarded |  |  |  |  |  |
| 39. | I become more easily excited by positive outcomes than other people |  |  |  |  |  |
