# Supplementary material for: The reward and punishment responsivity and motivation questionnaire (RPRM-Q): A stimulus-independent self-report measure of reward and punishment sensitivity that differentiates between responsivity and motivation
Source: Front Psychol. 2022 Aug 10;13:929255. doi: 10.3389/fpsyg.2022.929255 (PMC9404870; doi:10.3389/fpsyg.2022.929255)
Supplement: Supplementary file 2 [file Table_2.DOCX]

**Reward responsiveness**

| Original 12 items on reward responsivity | | |
| --- | --- | --- |
| **Nr in Q** | **Nr in MPlus** | **Question** |
| 1 | 1 | Winning makes me enthusiastic |
| 7 | 2 | Obtaining reward is very important to me |
| 12 | 3 | Positive outcomes motivate me strongly |
| 13 | 4 | Obtaining rewards affects me strongly |
| 14 | 5 | When I’m good at something, I like to keep at it |
| 21 | 6 | When something good happens, it affects me more strongly than others |
| 22 | 7 | When I achieve something I want, I feel excited and energized |
| 33 | 8 | Doing things I like makes me very happy |
| 34 | 9 | I can really enjoy nice things |
| 35 | 10 | When good things happen to me it affects me strongly |
| 38 | 11 | I like to be rewarded |
| 39 | 12 | I become more easily excited by positive outcomes than other people |

**Factor analysis 1 to 4 factors on 12 items**

1 factor solution RMSEA = 0.203, CFI = 0.838
2 factor solution RMSEA = 0.109, CFI = 0.963
3 factor solution RMSEA = 0.091, CFI = 0.980
4 factor solution RMSEA = 0.030, CFI = 0.998

| 4 factor EFA on 12 items | | | | |
| --- | --- | --- | --- | --- |
|  | **1** | **2** | **3** | **4** |
| 1 | 0.109 | 0.637 | -0.155 | 0.036 |
| 2 | 0.753 | -0.061 | -0.156 | 0.057 |
| 3 | 0.314 | 0.497 | 0.075 | -0.009 |
| 4 | 0.860 | 0.090 | 0.084 | -0.048 |
| 5 | 0.196 | 0.406 | 0.180 | -0.068 |
| 6 | -0.111 | 0.473 | -0.011 | 0.344 |
| 7 | -0.013 | 0.814 | 0.094 | -0.003 |
| 8 | 0.076 | 0.028 | 0.846 | -0.005 |
| 9 | -0.049 | -0.050 | 0.939 | 0.021 |
| 10 | 0.002 | 0.283 | 0.549 | 0.118 |
| 11 | 0.601 | -0.018 | 0.072 | 0.187 |
| 12 | 0.016 | -0.003 | 0.015 | 1.138 |

The fourth factor that was found in this analysis seems to hold the only two items that ask about people’s sensitivity relatively to that of others: “When something good happens, it affects me more strongly than others” & “I become more easily excited by positive outcomes than other people”. Since this might measure something else than reward responsivity, we excluded these two items. Additionally, items 2 and 5 were deleted because in hindsight these are not about reward responsivity.

**Factor analysis 1 to 4 factors on 8 items**

1 factor solution RMSEA = 0.233, CFI = 0.869
2 factor solution RMSEA = 0.100, CFI = 0.984
3 factor solution RMSEA = 0.000, CFI = 1.000
4 factor solution RMSEA = 0.000, CFI = 1.000

| 1 factor EFA on 8 items | |
| --- | --- |
|  | **1** |
| 1 | 0.527 |
| 3 | 0.701 |
| 4 | 0.641 |
| 7 | 0.737 |
| 8 | 0.810 |
| 9 | 0.786 |
| 10 | 0.716 |
| 11 | 0.503 |

| 2 factor EFA on 8 items | | |
| --- | --- | --- |
|  | **1** | **2** |
| 1 | 0.551 | 0.071 |
| 3 | 0.709 | 0.111 |
| 4 | 0.850 | -0.132 |
| 7 | 0.582 | 0.328 |
| 8 | 0.078 | 0.829 |
| 9 | -0.114 | 0.961 |
| 10 | 0.282 | 0.641 |
| 11 | 0.621 | -0.044 |

| 3 factor EFA on 8 items | | | |
| --- | --- | --- | --- |
|  | **1** | **2** | **3** |
| 1 | 0.715 | -0.017 | -0.135 |
| 3 | 0.619 | 0.184 | 0.027 |
| 4 | -0.003 | 1.294 | 0.000 |
| 7 | 0.785 | -0.026 | 0.124 |
| 8 | 0.076 | 0.041 | 0.814 |
| 9 | -0.082 | -0.020 | 0.971 |
| 10 | 0.333 | 0.019 | 0.575 |
| 11 | 0.303 | 0.312 | 0.023 |

| 4 factor EFA on 8 items | | | | |
| --- | --- | --- | --- | --- |
|  | **1** | **2** | **3** | **4** |
| 1 | -0.013 | 0.690 | -0.103 | 0.095 |
| 3 | 0.771 | 0.140 | 0.045 | -0.058 |
| 4 | 0.557 | -0.052 | -0.024 | 0.425 |
| 7 | 0.144 | 0.627 | 0.186 | 0.008 |
| 8 | 0.081 | -0.013 | 0.832 | 0.032 |
| 9 | -0.080 | -0.030 | 0.966 | -0.006 |
| 10 | 0.133 | 0.199 | 0.605 | 0.022 |
| 11 | -0.020 | 0.073 | 0.041 | 0.811 |

Item 11 is in the 4 factor solution an item on its own (together with 4 but that loads stronger on 1). This item is also content wise perhaps not the best fit, since it is not really asking about how responsive an individual is to reward, but how much they like to receive it.

**Factor analysis 1 to 4 factors on 7 items**

1 factor solution RMSEA = 0.242, CFI = 0.887
2 factor solution RMSEA = 0.063, CFI = 0.996
3 factor solution RMSEA = 0.000, CFI = 1.000

| 1 factor EFA on 7 items | |
| --- | --- |
|  | **1** |
| 1 | 0.519 |
| 3 | 0.702 |
| 4 | 0.574 |
| 7 | 0.743 |
| 8 | 0.826 |
| 9 | 0.804 |
| 10 | 0.728 |

| 2 factor EFA on 7 items | | |
| --- | --- | --- |
|  | **1** | **2** |
| 1 | 0.593 | -0.008 |
| 3 | 0.837 | -0.021 |
| 4 | 0.723 | -0.102 |
| 7 | 0.658 | 0.236 |
| 8 | 0.104 | 0.809 |
| 9 | -0.106 | 0.978 |
| 10 | 0.321 | 0.604 |

| 3 factor EFA on 7 items | | | |
| --- | --- | --- | --- |
|  | **1** | **2** | **3** |
| 1 | 0.043 | 0.663 | -0.137 |
| 3 | 0.699 | 0.142 | 0.042 |
| 4 | 0.791 | -0.065 | -0.015 |
| 7 | 0.012 | 0.799 | 0.098 |
| 8 | 0.113 | -0.012 | 0.826 |
| 9 | -0.086 | -0.016 | 0.967 |
| 10 | 0.140 | 0.209 | 0.586 |

Item 8 and 9 are the main reason for the second factor in the 2 factor solution. Also in the 3 factor solution they are the main reason for the third factor. They thus do not go well with the other items and are therefore excluded.

**Factor analysis 1 to 4 factors on 5 items**

1 factor solution RMSEA = 0.112, CFI = 0.981
2 factor solution RMSEA = 0.000, CFI = 1.000

| 1 factor EFA on 5 items | |
| --- | --- |
|  | **1** |
| 1 | 0.589 |
| 3 | 0.813 |
| 4 | 0.652 |
| 7 | 0.796 |
| 10 | 0.600 |

| 2 factor EFA on 5 items | | |
| --- | --- | --- |
|  | **1** | **2** |
| 1 | 0.308 | 0.311 |
| 3 | 0.882 | 0.003 |
| 4 | 0.689 | -0.018 |
| 7 | -0.008 | 1.041 |
| 10 | 0.271 | 0.355 |

The one factor solution is still not really good. The two factor solution shows that this is due to item 7 which is therefore excluded.

**Factor analysis 1 to 4 factors on 4items**

1 factor solution RMSEA = 0.000, CFI = 1.000

| 1 factor EFA on 4 items | |
| --- | --- |
|  | **1** |
| 1 | 0.528 |
| 3 | 0.876 |
| 4 | 0.675 |
| 10 | 0.517 |

**Motivation to approach reward**

| Original 9 items on motivation to approach reward | | |
| --- | --- | --- |
| **Nr in Q** | **Nr in MPlus** | **Question** |
| 3 | 1 | When I want something I usually go all-out to get it |
| 5 | 2 | I go out of my way to get things I want |
| 6 | 3 | When I am doing something I like I do not consider the consequences |
| 10 | 4 | I am more inclined to work hard to get positive outcomes than others |
| 15 | 5 | If I see a chance to get something I want I move on it right away |
| 16 | 6 | When I see something I want I will do everything necessary to obtain it |
| 18 | 7 | I work hard for things that are potentially rewarding for me |
| 24 | 8 | I always try to get things I want, even if it means I have to work hard for it |
| 30 | 9 | If I can obtain reward I am very motivated to give it my all |

**Factor analysis 1 to 4 factors on 9 items**

1 factor solution RMSEA = 0.097, CFI = 0.969
2 factor solution RMSEA = 0.065, CFI = 0.990
3 factor solution RMSEA = 0.000, CFI = 1.000
4 factor solution RMSEA = 0.000, CFI = 1.000

| 1 factor EFA on 9 items | |
| --- | --- |
|  | **1** |
| 1 | 0.729 |
| 2 | 0.635 |
| 3 | -0.180 |
| 4 | 0.684 |
| 5 | 0.656 |
| 6 | 0.736 |
| 7 | 0.608 |
| 8 | 0.753 |
| 9 | 0.502 |

| 2 factor EFA on 9 items | | |
| --- | --- | --- |
|  | **1** | **2** |
| 1 | 0.368 | 0.434 |
| 2 | 0.288 | 0.413 |
| 3 | -0.482 | 0.286 |
| 4 | 0.756 | -0.016 |
| 5 | 0.150 | 0.566 |
| 6 | -0.037 | 0.882 |
| 7 | 0.579 | 0.084 |
| 8 | 0.719 | 0.102 |
| 9 | 0.189 | 0.369 |

| 3 factor EFA on 9 items | | | |
| --- | --- | --- | --- |
|  | **1** | **2** | **3** |
| 1 | 0.362 | 0.462 | -0.009 |
| 2 | 0.289 | 0.467 | -0.066 |
| 3 | -0.467 | 0.237 | 0.042 |
| 4 | 0.709 | -0.013 | 0.088 |
| 5 | 0.140 | 0.488 | 0.152 |
| 6 | -0.041 | 0.863 | 0.054 |
| 7 | 0.565 | -0.035 | 0.225 |
| 8 | 0.733 | 0.155 | -0.068 |
| 9 | 0.009 | 0.023 | 0.971 |

| 4 factor EFA on 9 items | | | | |
| --- | --- | --- | --- | --- |
|  | **1** | **2** | **3** | **4** |
| 1 | 0.654 | 0.088 | 0.084 | -0.132 |
| 2 | 0.638 | 0.008 | 0.071 | -0.185 |
| 3 | 0.064 | -0.199 | -0.146 | 0.187 |
| 4 | -0.011 | 0.963 | -0.016 | 0.020 |
| 5 | 0.557 | 0.021 | 0.132 | 0.170 |
| 6 | 0.868 | -0.022 | -0.082 | 0.128 |
| 7 | -0.010 | -0.010 | 0.993 | 0.012 |
| 8 | 0.422 | 0.309 | 0.163 | -0.202 |
| 9 | 0.213 | 0.153 | 0.227 | 0.441 |

Item 3 does not fit well with the other items. This item refers specifically to consequences, and its might fit better as a reversed item of the motivation to avoid punishment scale. In hindsight we feel that item 8 is more a measure of persistency or perseverance. Question 3 and 8 were therefore excluded. Question 3 will be added to the motivation to avoid punishment analysis.

**Factor analysis 1 to 4 factors on 7 items**

1 factor solution RMSEA = 0.090, CFI = 0.979
2 factor solution RMSEA = 0.075, CFI = 0.992
3 factor solution RMSEA = 0.000, CFI = 1.000

| 1 factor EFA on 7 items | |
| --- | --- |
|  | **1** |
| 1 | 0.734 |
| 2 | 0.639 |
| 4 | 0.614 |
| 5 | 0.674 |
| 6 | 0.759 |
| 7 | 0.589 |
| 9 | 0.538 |

| 2 factor EFA on 7 items | | |
| --- | --- | --- |
|  | **1** | **2** |
| 1 | 0.619 | 0.175 |
| 2 | 0.557 | 0.131 |
| 4 | 0.334 | 0.375 |
| 5 | 0.569 | 0.157 |
| 6 | 0.906 | -0.112 |
| 7 | -0.001 | 0.824 |
| 9 | 0.302 | 0.321 |

| 3 factor EFA on 7 items | | | |
| --- | --- | --- | --- |
|  | **1** | **2** | **3** |
| 1 | 0.655 | 0.174 | -0.046 |
| 2 | 0.656 | 0.124 | -0.139 |
| 4 | 0.551 | -0.010 | 0.187 |
| 5 | 0.190 | 0.415 | 0.235 |
| 6 | 0.050 | 0.902 | 0.004 |
| 7 | 0.505 | -0.118 | 0.417 |
| 9 | -0.013 | 0.326 | 0.513 |

Item 6 seems to be the reason for three factors in the 3 factor solution, and it has a negative loading in the 2 factor solution. Therefore, item 6 was deleted.

**Factor analysis 1 to 4 factors on 6 items**

1 factor solution RMSEA = 0.072, CFI = 0.985
2 factor solution RMSEA = 0.000, CFI = 1.000

| 1 factor EFA on 6 items | |
| --- | --- |
|  | **1** |
| 1 | 0.728 |
| 2 | 0.624 |
| 4 | 0.643 |
| 5 | 0.631 |
| 7 | 0.640 |
| 9 | 0.534 |

| 2 factor EFA on 6 items | | |
| --- | --- | --- |
|  | **1** | **2** |
| 1 | 0.742 | 0.042 |
| 2 | 0.728 | -0.063 |
| 4 | 0.397 | 0.300 |
| 5 | 0.329 | 0.366 |
| 7 | 0.224 | 0.492 |
| 9 | -0.064 | 0.714 |

The 1 factor solution is already sufficient. Yet, if we want to end up with 5 items, item 9 is the best to delete.

**Factor analysis 1 to 4 factors on 5 items**

1 factor solution RMSEA = 0.040, CFI = 0.997

| 1 factor EFA on 5 items | |
| --- | --- |
|  | **1** |
| 1 | 0.753 |
| 2 | 0.650 |
| 4 | 0.640 |
| 5 | 0.604 |
| 7 | 0.608 |

**Punishment Responsivity**

| Original 10 items on punishment responsivity | | |
| --- | --- | --- |
| **Nr in Q** | **Nr in MPlus** | **Question** |
| 2 | 1 | Losing gives me a bad feeling |
| 8 | 2 | When I do something poorly it affects me strongly |
| 11 | 3 | I really dislike being rejected |
| 19 | 4 | Criticism or scolding hurts me a lot |
| 20 | 5 | When something unpleasant happens I get pretty “worked up” about it |
| 23 | 6 | I feel lousy after doing something wrong |
| 28 | 7 | When someone points out I did something wrong I feel miserable |
| 29 | 8 | Receiving punishment affects me strongly |
| 32 | 9 | I feel really bad when something negative happens to me |
| 36 | 10 | Negative outcomes affect me more strongly than others |

**Factor analysis 1 to 4 factors on 10 items**

1 factor solution RMSEA = 0.092, CFI = 0.978
2 factor solution RMSEA = 0.081, CFI = 0.987
3 factor solution not provided
4 factor solution RMSEA = 0.068, CFI = 0.996

| 1 factor EFA on 10 items | |
| --- | --- |
|  | **1** |
| 1 | 0.262 |
| 2 | 0.461 |
| 3 | 0.580 |
| 4 | 0.759 |
| 5 | 0.695 |
| 6 | 0.708 |
| 7 | 0.772 |
| 8 | 0.744 |
| 9 | 0.667 |
| 10 | 0.649 |

| 2 factor EFA on 10 items | | |
| --- | --- | --- |
|  | **1** | **2** |
| 1 | 0.384 | -0.107 |
| 2 | 0.654 | -0.160 |
| 3 | 0.331 | 0.288 |
| 4 | 0.348 | 0.458 |
| 5 | 0.715 | 0.027 |
| 6 | 0.541 | 0.219 |
| 7 | 0.015 | 0.821 |
| 8 | -0.008 | 0.815 |
| 9 | 0.434 | 0.276 |
| 10 | 0.648 | 0.041 |

| 3 factor EFA on 10 items | | | | |
| --- | --- | --- | --- | --- |
|  | **1** | **2** | **3** | **4** |
| 1 | 0.175 | 0.047 | 0.229 | -0.095 |
| 2 | 0.044 | 0.034 | 0.638 | -0.050 |
| 3 | 0.499 | -0.075 | 0.238 | 0.015 |
| 4 | 0.893 | 0.013 | -0.056 | 0.047 |
| 5 | 0.431 | 0.100 | 0.330 | 0.014 |
| 6 | -0.016 | 0.031 | 0.521 | 0.381 |
| 7 | 0.000 | 0.017 | -0.019 | 0.872 |
| 8 | 0.127 | -0.004 | 0.027 | 0.675 |
| 9 | 0.314 | 0.101 | 0.165 | 0.229 |
| 10 | -0.001 | 1.674 | -0.001 | -0.001 |

Item 10 seems to be a factor on its own. This is again an item that ask about sensitivity in relation to others which might measure something else than we want to. This item is therefore deleted.

**Factor analysis 1 to 4 factors on 9 items**

1 factor solution RMSEA = 0.088, CFI = 0.982

2 factor solution RMSEA = 0.080, CFI = 0.989

3 factor solution RMSEA = 0.060, CFI = 0.996

| 1 factor EFA on 9 items | |
| --- | --- |
|  | **1** |
| 1 | 0.254 |
| 2 | 0.454 |
| 3 | 0.602 |
| 4 | 0.765 |
| 5 | 0.676 |
| 6 | 0.706 |
| 7 | 0.776 |
| 8 | 0.756 |
| 9 | 0.650 |

| 2 factor EFA on 9 items | | |
| --- | --- | --- |
|  | **1** | **2** |
| 1 | 0.331 | -0.034 |
| 2 | 0.681 | -0.125 |
| 3 | 0.389 | 0.279 |
| 4 | 0.277 | 0.547 |
| 5 | 0.513 | 0.247 |
| 6 | 0.472 | 0.322 |
| 7 | -0.031 | 0.851 |
| 8 | -0.024 | 0.820 |
| 9 | 0.290 | 0.417 |

| 3 factor EFA on 9 items | | | |
| --- | --- | --- | --- |
|  | **1** | **2** | **3** |
| 1 | 0.178 | 0.244 | -0.096 |
| 2 | 0.044 | 0.645 | -0.060 |
| 3 | 0.476 | 0.226 | 0.009 |
| 4 | 0.899 | -0.051 | 0.047 |
| 5 | 0.438 | 0.364 | 0.013 |
| 6 | -0.020 | 0.547 | 0.365 |
| 7 | 0.003 | -0.015 | 0.875 |
| 8 | 0.130 | 0.032 | 0.667 |
| 9 | 0.322 | 0.193 | 0.236 |

Item 1 does not seem to load on any factor and is therefore deleted.

**Factor analysis 1 to 4 factors on 8 items**

1 factor solution RMSEA = 0.101, CFI = 0.982

2 factor solution RMSEA = 0.099, CFI = 0.988

3 factor solution RMSEA = 0.078, CFI = 0.996

4 factor solution RMSEA = 0.000, CFI = 1.000

| 1 factor EFA on 8 items | |
| --- | --- |
|  | **1** |
| 2 | 0.448 |
| 3 | 0.602 |
| 4 | 0.764 |
| 5 | 0.678 |
| 6 | 0.704 |
| 7 | 0.780 |
| 8 | 0.759 |
| 9 | 0.648 |

| 2 factor EFA on 8 items | | |
| --- | --- | --- |
|  | **1** | **2** |
| 2 | 0.663 | -0.164 |
| 3 | 0.465 | 0.183 |
| 4 | 0.378 | 0.435 |
| 5 | 0.709 | 0.034 |
| 6 | 0.541 | 0.224 |
| 7 | -0.017 | 0.858 |
| 8 | 0.034 | 0.774 |
| 9 | 0.348 | 0.343 |

| 3 factor EFA on 8 items | | | |
| --- | --- | --- | --- |
|  | **1** | **2** | **3** |
| 2 | 0.111 | -0.089 | 0.549 |
| 3 | 0.547 | -0.009 | 0.144 |
| 4 | 0.820 | 0.125 | -0.082 |
| 5 | 0.522 | -0.095 | 0.372 |
| 6 | -0.017 | 0.235 | 0.695 |
| 7 | 0.022 | 0.857 | 0.037 |
| 8 | 0.205 | 0.566 | 0.081 |
| 9 | 0.385 | 0.199 | 0.154 |

| 4 factor EFA on 8 items | | | | |
| --- | --- | --- | --- | --- |
|  | **1** | **2** | **3** | **4** |
| 2 | 0.614 | 0.164 | 0.040 | -0.005 |
| 3 | 0.054 | 0.856 | -0.015 | 0.008 |
| 4 | -0.139 | 0.309 | 0.187 | 0.505 |
| 5 | 0.003 | -0.009 | 1.194 | -0.009 |
| 6 | 0.392 | -0.040 | 0.131 | 0.470 |
| 7 | 0.027 | -0.035 | -0.049 | 0.889 |
| 8 | -0.016 | 0.043 | 0.023 | 0.761 |
| 9 | 0.049 | 0.215 | 0.098 | 0.417 |

Items 5 is a factor on its own in the 4 factor solution. The item is about *unpleasant things*, and getting *worked up about it*. The formulation of this item might not be clear enough and it is deleted.

Item 3 also seems to be a factor on its own in the 4 factor solution (together with 4 but that loads stronger on factor 4), as well as item 2 (together with 6 but that loads stronger on factor 4). Both items are deleted.

**Factor analysis 1 to 4 factors on 5 items**

1 factor solution RMSEA = 0.000, CFI = 1.000

| 1 factor EFA on 5 items | |
| --- | --- |
|  | **1** |
| 4 | 0.723 |
| 6 | 0.663 |
| 7 | 0.836 |
| 8 | 0.791 |
| 9 | 0.630 |

**Motivation to avoid Punishment**

| Original 9 items on motivation to avoid punishment | | |
| --- | --- | --- |
| **Nr in Q** | **Nr in MPlus** | **Question** |
| 4 | 1 | I work harder than others to avoid negative outcomes |
| 9 | 2 | I do everything in my power to avoid receiving punishment |
| 17 | 3 | If something fun seems to be taken away, I do everything I can to prevent this |
| 25 | 4 | If it seems that I will lose, I will go to extremes to avoid this |
| 26 | 5 | I go out of my way to avoid unpleasant things happening to me |
| 27 | 6 | I do everything I can to avoid receiving criticism |
| 31 | 7 | I avoid things that might have a negative outcome |
| 37 | 8 | I work hard to ensure I will not be rejected |
| 6 | 9 | When I am doing something I like I do not consider the consequences |

**Factor analysis 1 to 4 factors on 9 items**

1 factor solution RMSEA = 0.134, CFI = 0.852

2 factor solution RMSEA = 0.121, CFI = 0.914

3 factor solution RMSEA = 0.096, CFI = 0.966

4 factor solution RMSEA = 0.049, CFI = 0.996

| 1 factor EFA on 9 items | |
| --- | --- |
|  | **1** |
| 1 | 0.302 |
| 2 | 0.622 |
| 3 | 0.174 |
| 4 | 0.414 |
| 5 | 0.610 |
| 6 | 0.589 |
| 7 | 0.646 |
| 8 | 0.604 |
| 9 | -0.143 |

| 2 factor EFA on 9 items | | |
| --- | --- | --- |
|  | **1** | **2** |
| 1 | 0.743 | -0.011 |
| 2 | 0.112 | 0.575 |
| 3 | -0.052 | 0.206 |
| 4 | 0.267 | 0.296 |
| 5 | 0.094 | 0.571 |
| 6 | -0.099 | 0.662 |
| 7 | -0.098 | 0.726 |
| 8 | 0.299 | 0.476 |
| 9 | -0.374 | 0.045 |

| 3 factor EFA on 9 items | | | |
| --- | --- | --- | --- |
|  | **1** | **2** | **3** |
| 1 | -0.032 | 0.222 | 0.533 |
| 2 | 0.545 | 0.075 | 0.121 |
| 3 | 0.106 | 0.271 | -0.212 |
| 4 | -0.001 | 0.787 | 0.031 |
| 5 | 0.449 | 0.327 | -0.036 |
| 6 | 0.710 | -0.090 | -0.034 |
| 7 | 0.697 | 0.059 | -0.097 |
| 8 | 0.509 | -0.021 | 0.388 |
| 9 | 0.034 | 0.073 | -0.513 |

| 4 factor EFA on 9 items | | | | |
| --- | --- | --- | --- | --- |
|  | **1** | **2** | **3** | **4** |
| 1 | 0.306 | -0.071 | 0.190 | -0.255 |
| 2 | 0.068 | 0.543 | 0.060 | -0.128 |
| 3 | 0.281 | 0.005 | 0.092 | 0.335 |
| 4 | 0.784 | 0.017 | 0.004 | 0.014 |
| 5 | 0.292 | 0.500 | -0.048 | -0.051 |
| 6 | -0.083 | 0.585 | 0.150 | 0.092 |
| 7 | -0.028 | 0.769 | -0.031 | 0.012 |
| 8 | 0.000 | 0.004 | 1.267 | -0.001 |
| 9 | 0.007 | -0.008 | -0.017 | 0.725 |

Item 8 seems to be a factor on its own in the 4 factor solution. This item is about rejection. In the punishment responsivity subscale this item was also deleted, so also for sake of consistency it is good to delete it.

**Factor analysis 1 to 4 factors on 8 items**

1 factor solution RMSEA = 0.128, CFI = 0.854

2 factor solution RMSEA = 0.115, CFI = 0.924

3 factor solution RMSEA = 0.034, CFI = 0.996

4 factor solution RMSEA = 0.000, CFI = 1.000

| 1 factor EFA on 8 items | |
| --- | --- |
|  | **1** |
| 1 | 0.232 |
| 2 | 0.613 |
| 3 | 0.180 |
| 4 | 0.431 |
| 5 | 0.661 |
| 6 | 0.546 |
| 7 | 0.671 |
| 9 | -0.093 |

| 2 factor EFA on 8 items | | |
| --- | --- | --- |
|  | **1** | **2** |
| 1 | 0.548 | -0.079 |
| 2 | 0.171 | 0.521 |
| 3 | 0.042 | 0.161 |
| 4 | 0.499 | 0.165 |
| 5 | 0.290 | 0.501 |
| 6 | -0.108 | 0.647 |
| 7 | -0.041 | 0.749 |
| 9 | -0.360 | 0.129 |

| 3 factor EFA on 8 items | | | |
| --- | --- | --- | --- |
|  | **1** | **2** | **3** |
| 1 | 0.014 | 0.296 | -0.259 |
| 2 | 0.579 | 0.054 | -0.134 |
| 3 | 0.058 | 0.253 | 0.274 |
| 4 | -0.001 | 0.843 | 0.011 |
| 5 | 0.487 | 0.267 | -0.043 |
| 6 | 0.634 | -0.071 | 0.061 |
| 7 | 0.758 | 0.033 | 0.033 |
| 9 | 0.000 | 0.009 | 0.823 |

| 4 factor EFA on 8 items | | | | |
| --- | --- | --- | --- | --- |
|  | **1** | **2** | **3** | **4** |
| 1 | 0.139 | 0.023 | 0.075 | -0.338 |
| 2 | -0.033 | 0.482 | 0.243 | -0.253 |
| 3 | 0.051 | -0.049 | 0.541 | 0.197 |
| 4 | 1.481 | 0.000 | -0.001 | 0.000 |
| 5 | 0.110 | 0.447 | 0.210 | -0.149 |
| 6 | -0.034 | 0.566 | 0.083 | 0.038 |
| 7 | 0.017 | 0.846 | -0.093 | 0.065 |
| 9 | -0.005 | 0.052 | 0.092 | 0.687 |

Item 9 seems to be a factor on its own. So it seems not to have been a good decision to add it to this scale.

**Factor analysis 1 to 4 factors on 7 items**

1 factor solution RMSEA = 0.117, CFI = 0.911

2 factor solution RMSEA = 0.038, CFI = 0.995

| 1 factor EFA on 7 items | |
| --- | --- |
|  | **1** |
| 1 | 0.221 |
| 2 | 0.608 |
| 3 | 0.190 |
| 4 | 0.429 |
| 5 | 0.659 |
| 6 | 0.550 |
| 7 | 0.676 |

| 2 factor EFA on 7 items | | |
| --- | --- | --- |
|  | **1** | **2** |
| 1 | 0.290 | 0.043 |
| 2 | 0.057 | 0.584 |
| 3 | 0.187 | 0.068 |
| 4 | 0.914 | -0.002 |
| 5 | 0.246 | 0.505 |
| 6 | -0.081 | 0.628 |
| 7 | -0.038 | 0.755 |

Item 4 seems to be the main reason for the second factor in the 2 factor solution. This item is about *going to extremes* and might be a too extreme form of approach behavior for the goal of the questionnaire.

**Factor analysis 1 to 4 factors on 6 items**

1 factor solution RMSEA = 0.040, CFI = 0.991

2 factor solution RMSEA = 0.000, CFI = 1.000

| 1 factor EFA on 6 items | |
| --- | --- |
|  | **1** |
| 1 | 0.163 |
| 2 | 0.624 |
| 3 | 0.146 |
| 5 | 0.598 |
| 6 | 0.586 |
| 7 | 0.724 |

| 2 factor EFA on 6 items | | |
| --- | --- | --- |
|  | **1** | **2** |
| 1 | 0.247 | -0.080 |
| 2 | 0.658 | -0.008 |
| 3 | 0.255 | -0.115 |
| 5 | 0.635 | -0.011 |
| 6 | 0.410 | 0.168 |
| 7 | 0.002 | 1.133 |

Item 1 and 3 do not load on a one factor solution, and still not when item 7, which apparently can be a factor on its own, is not included. Therefore items 1 and 3 were deleted.

**Factor analysis 1 to 4 factors on 4 items**

1 factor solution RMSEA = 0.052, CFI = 0.996

| 1 factor EFA on 4 items | |
| --- | --- |
|  | **1** |
| 2 | 0.610 |
| 5 | 0.582 |
| 6 | 0.591 |
| 7 | 0.745 |

**RPRM-Q**

| Selected questions for final RPRM questionnaire | | |
| --- | --- | --- |
| *Reward responsivity* | | |
| **Nr in Q** | **Nr in MPlus** | **Question** |
| 1 | 1 | Winning makes me enthusiastic |
| 12 | 2 | Positive outcomes motivate me strongly |
| 13 | 3 | Obtaining rewards affects me strongly |
| 35 | 4 | When good things happen to me it affects me strongly |
| *Motivation to approach reward* | | |
| **Nr in Q** | **Nr in MPlus** | **Question** |
| 3 | 5 | When I want something I usually go all-out to get it |
| 5 | 6 | I go out of my way to get things I want |
| 10 | 7 | I am more inclined to work hard to get positive outcomes than others |
| 15 | 8 | If I see a chance to get something I want I move on it right away |
| 18 | 9 | I work hard for things that are potentially rewarding for me |
| *Punishment responsivity* | | |
| **Nr in Q** | **Nr in MPlus** | **Question** |
| 19 | 10 | Criticism or scolding hurts me a lot |
| 23 | 11 | I feel lousy after doing something wrong |
| 28 | 12 | When someone points out I did something wrong I feel miserable |
| 29 | 13 | Receiving punishment affects me strongly |
| 32 | 14 | I feel really bad when something negative happens to me |
| *Motivation to avoid punishment* | | |
| **Nr in Q** | **Nr in MPlus** | **Question** |
| 9 | 15 | I do everything in my power to avoid receiving punishment |
| 26 | 16 | I go out of my way to avoid unpleasant things happening to me |
| 27 | 17 | I do everything I can to avoid receiving criticism |
| 31 | 18 | I avoid things that might have a negative outcome |

**Factor analysis 4 factors on 18 items**

CFI = 0.995, RMSEA = 0.034

| 4 factor EFA on 18 items | | | | |
| --- | --- | --- | --- | --- |
|  | **1** | **2** | **3** | **4** |
| 1 | 0.466 | 0.090 | 0.065 | 0.005 |
| 2 | 0.865 | 0.050 | 0.030 | -0.089 |
| 3 | 0.596 | 0.078 | 0.057 | 0.243 |
| 4 | 0.589 | -0.095 | -0.040 | -0.026 |
| 5 | 0.110 | 0.684 | -0.069 | 0.010 |
| 6 | -0.074 | 0.770 | 0.084 | -0.144 |
| 7 | 0.024 | 0.592 | -0.006 | 0.132 |
| 8 | 0.289 | 0.421 | -0.182 | 0.173 |
| 9 | 0.298 | 0.425 | -0.034 | 0.162 |
| 10 | 0.008 | 0.043 | 0.674 | 0.097 |
| 11 | 0.177 | -0.088 | 0.634 | -0.019 |
| 12 | -0.090 | 0.061 | 0.893 | -0.029 |
| 13 | 0.107 | -0.079 | 0.752 | 0.026 |
| 14 | 0.171 | -0.004 | 0.425 | 0.297 |
| 15 | 0.128 | 0.044 | 0.117 | 0.552 |
| 16 | -0.074 | 0.163 | 0.097 | 0.509 |
| 17 | -0.126 | 0.014 | 0.467 | 0.308 |
| 18 | -0.062 | -0.101 | 0.064 | 0.729 |

Item 17 seems to fit better with punishment responsivity than with motivation to avoid punishment were it was intended. Inspection of this item “I do everything I can to avoid receiving criticism” shows that it correlates with two items of the punishment responsivity scale that have similar wording. Although the fit of the 4 factor solution would improve if item 17 would be added to punishment responsivity, based on the content it seem preferable to keep it with the motivation to avoid punishment subscale.

**Factor analysis 2 factors on 18 items**

CFI = 0.951, RMSEA = 0.090

| 2 factor EFA on 18 items | | |
| --- | --- | --- |
|  | **1** | **2** |
| 1 | 0.463 | 0.093 |
| 2 | 0.731 | 0.043 |
| 3 | 0.619 | 0.270 |
| 4 | 0.416 | 0.003 |
| 5 | 0.688 | -0.165 |
| 6 | 0.565 | -0.145 |
| 7 | 0.562 | 0.004 |
| 8 | 0.648 | -0.086 |
| 9 | 0.646 | 0.048 |
| 10 | 0.001 | 0.727 |
| 11 | 0.009 | 0.630 |
| 12 | -0.099 | 0.826 |
| 13 | -0.046 | 0.767 |
| 14 | 0.164 | 0.641 |
| 15 | 0.247 | 0.514 |
| 16 | 0.176 | 0.449 |
| 17 | -0.060 | 0.669 |
| 18 | 0.021 | 0.586 |

A last check was the 2 factor solution of the 18 items. This solution has an acceptable fit and show a reward sensitivity and a punishment sensitivity subscale.
