# Supplementary material for: The reward and punishment responsivity and motivation questionnaire (RPRM-Q): A stimulus-independent self-report measure of reward and punishment sensitivity that differentiates between responsivity and motivation
Source: Front Psychol. 2022 Aug 10;13:929255. doi: 10.3389/fpsyg.2022.929255 (PMC9404870; doi:10.3389/fpsyg.2022.929255)
Supplement: Supplementary file 3 [file Table_3.docx]

**Appendix**

The Reward and Punishment Responsivity and motivation to approach or avoid questionnaire.

This questionnaire consists of 18 items. Please read each statement carefully and indicate how much it applies to you.

|  | **This applies to me completely** | **This applies to me a bit** | **Neutral** | **This does not really apply to me** | **This does not apply to me at all** |
| --- | --- | --- | --- | --- | --- |
| 1. Winning makes me enthusiastic |  |  |  |  |  |
| 2. When I want something I usually go all-out to get it |  |  |  |  |  |
| 3. I go out of my way to get things I want |  |  |  |  |  |
| 4. I do everything in my power to avoid receiving punishment |  |  |  |  |  |
| 5. I am more inclined to work hard to get positive outcomes than others |  |  |  |  |  |
| 6. Positive outcomes motivate me strongly |  |  |  |  |  |
| 7. Obtaining rewards affects me strongly |  |  |  |  |  |
| 8. If I see a chance to get something I want I move on it right away |  |  |  |  |  |
| 9. I work hard for things that are potentially rewarding for me |  |  |  |  |  |
| 10. Criticism or scolding hurts me a lot |  |  |  |  |  |
| 11. I feel lousy after doing something wrong |  |  |  |  |  |
| 12. I go out of my way to avoid unpleasant things happening to me |  |  |  |  |  |
| 13. I do everything I can to avoid receiving criticism |  |  |  |  |  |
| 14. When someone points out I did something wrong I feel miserable |  |  |  |  |  |
| 15. Receiving punishment affects me strongly |  |  |  |  |  |
| 16. I avoid things that might have a negative outcome |  |  |  |  |  |
| 17. I feel really bad when something negative happens to me |  |  |  |  |  |
| 18. When good things happen to me it affects me strongly |  |  |  |  |  |
